# Dynamic analysis of geomaterials using microwave sensing

**DOI:** 10.1038/s41598-024-57653-3

**Published:** 2024-03-26

**Authors:** Jamie Blanche, Daniel Mitchell, Junlong Shang, David Flynn, Sumanth Pavuluri, Marc Desmulliez

**Affiliations:** 1 James Watt School of Engineering, University of Glasgow, Glasgow, UK; 2 Department of Mechanical, Aerospace & Civil Engineering, University of Manchester, Manchester, UK; 3 Smart Systems Group, School of Engineering and Physical Sciences, Heriot-Watt University, Edinburgh, UK

**Keywords:** Solid Earth sciences, Geodynamics, Geology, Petrology, Techniques and instrumentation, Characterization and analytical techniques

## Abstract

Precise characterization of geomaterials improves subsurface energy extraction and storage. Understanding geomaterial property, and the complexities between petrophysics and geomechanics, plays a key role in maintaining energy security and the transition to a net zero global carbon economy. Multiple sectors demand accurate and rapid characterization of geomaterial conditions, requiring the extraction of core plugs in the field for full-field characterization and analysis in the laboratory. We present a novel technique for the non-invasive characterization of geomaterials by using Frequency Modulated Continuous Wave (FMCW) radar in the K-band, representing a new application of microwave radar. We collect data through the delivery of FMCW wave interactions with geomaterials under static and dynamic conditions and show that FMCW can detect fluid presence, differentiate fluid type, indicate the presence of metallic inclusions and detect imminent failure in loaded sandstones by up to 15 s, allowing for greater control in loading up to a failure event. Such precursors have the potential to significantly enhance our understanding of, and ability to model, geomaterial dynamics. This low-cost sensing method is easily deployable, provides quicker and more accessible data than many state-of-the-art systems, and new insights into geomaterial behavior under dynamic conditions.

## Introduction

The global consumption of fossil fuels is a major driver in the creation of major greenhouse gases, such as CO_2_ and CH_4_, and polluted water produced by drilling and mining activities^1–4^. Understanding the dynamic geomechanical conditions of the subsurface, and the motion of fluids through reservoir rock units, is a common requirement for all sectors that exploit the subsurface. The research presented in this paper addresses the multi-sectoral need to safeguard human subsurface activities, while also improving efficiency of operations and modelling.

The dynamic loading of subsurface reservoir environments, whether natural or due to human activity, can result in pressure gradients and fluid movement, in addition to rock deformation and failure events, which alter the flow pathways of fluid fronts and may result in the motion of hazardous materials from the subsurface into the environment^5–7^. These are often unseen events that are nevertheless likely to result in significant socio-economic impacts.

The study of reservoir rocks and their sealing or permeable properties has developed in tandem with the historical progression of the petroleum industry, with a strong focus on how fluids move between stratigraphic layers for extraction or containment^8–17^. The body of knowledge gained from the petroleum sector has been transferred to the rise of geologic CO_2_ storage in response to environmental requirements and the global obligation to act towards decarbonization and Net Zero^18–22^. The proliferation of non-fossil fuel energy generation and mining enterprises has also led to the translation of reservoir geology to geothermal energy extraction^23–25^ and nuclear waste disposal^26,27^. Thus, the characterization of the subsurface is a key component of subsurface process management, necessitating description and quantification of petrophysical parameters, such as porosity, permeability and the presence and magnitude of fracturing events.

Similarly, the geo-resource extraction, subsurface carbon waste storage and civil infrastructure sectors are heavily reliant on the same geomechanical knowledge base and operational techniques to succeed. This requires knowledge of the rock mineralogical configuration, and the movement of one or more fluid phases through rock mass pore systems. These sectors are primarily concerned with rock damage, natural or by human influence, and the dominant fluid flow pathways through altered rock units. The importance of rock integrity and reservoir systems elements mapping across multiple key energy sectors are well defined for oil and gas (O&G)^28,29^, carbon capture and storage (CCS)^30–36^, geothermal energy extraction^37–40^, nuclear waste disposal^26,41–43^ and mining operations^44–46^. For example, the generation of nuclear energy throughout the lifecycle of a powerplant creates extremely long-lived waste products, which must be reliably isolated from the geosphere and biosphere. Ultimately, high fidelity measurement of key subsurface geomaterial and dynamic structural measurands is at the core of many global environmental issues, where low-cost economy, accuracy and deployability are highly desirable traits in sensor development.

Development of the knowledge base required to advance characterization of subsurface geomaterial is typically hampered by the difficulties in acquiring pore fluid distributions and their representation in 3D space. Advancements in imaging technology have however enabled the full-volume characterization of geomaterial samples, placing emphasis on mapping the 3D pore space system. These methods include X-ray tomography for multi-phasic fluid distribution, via the application of injected dopants acting as contrast agents ^47^, and where the use of a synchrotron can result in rapid X-ray tomography^48^. The imaging of fluid flow processes can be achieved via neutron tomography in a controlled environment^13,17^. The simultaneous use of neutron and X-ray tomographic methods can detect rock framework and fluid movement through the pore network in real time^49^.

The exploitation of subsurface resources depends on understanding both natural and human-induced fluid flow processes and how they affect the evolution of geomaterials, particularly at the pore scale^8–14,16,19,50,51^. Laboratory-based experimental studies are crucial in the development of geomaterial knowledge and the multi-phase fluids contained therein^15,17,52^. The application of experimentally attained data also plays a critical role in the continued refinement of numerical simulations of subsurface rocks and the dynamic reservoir environment, where even a 1% increase in modelling accuracy can result in significant savings in capital and operational expenditure^18,20,53^. Advances in subsurface modelling also result in more accuracy in rock damage prediction and reservoir systems element condition^54–57^, further improving the safety of subsurface operations via enhanced understanding of fluid movement, dynamic petrophysical and geomechanical properties^12^. Reliable predictions on fluid flow characteristics, the design of monitoring schemes and the development of more efficient operations, require a deeper understanding of the flow process, and how the flow relates to the intrinsic composition and structure of the rock^58^.

For both carbon storage and hydrocarbon resource extraction, porous reservoir rock sequences are overlain by sealing rocks, which are much less permeable than the adjacent stratigraphic layers, and which act as significant baffles to fluid exit or entry^59–63^. Sealing rocks, and the need to quantify their porosity, permeability, fracturing and faulting, are essential components of effective reservoir management and operation. Laboratory measurements and theoretical models are commonly used to derive estimates of the flow characteristics of any given rock, with those approaches often being extended to progress from the specific rock sample towards a generalization of the most significant factors in governing fluid flow behavior. Performing these measurements typically requires removal of material from the reservoir site to a laboratory setting for analysis; an environment not wholly representative of the subsurface and where additional factors may affect the true accuracy of the measurement. Our research focus is on improving the accuracy of these measurements, resulting in increased operational efficiency for the subsurface energy generation or waste storage sectors via more accurate modelling techniques.

Table 1 shows a comparison of literature since 2018 for microwave sensing applied to geomaterials and detection of fluids within porous media, with a focus on the frequency band, antenna type, measurand and whether the datasets have been utilized within Artificial Intelligence (AI) or Machine Learning (ML) processes. Notable similarities exist between Adbolrazzaghi et al.^64^, Kazemi et al.^65^, Ferhat et al.^66^, Shen et al.^67^, Alvarez et al.^68^ and Orr et al.^69^, all operating within a 0.17–8.6 GHz frequency range, representing a wavelength range of 3.5–176 cm. Of these, Ferhat et al., Shen et al., and Alvarez et al., provide insight into fluid presence detection within geomaterials and analogues, such as building materials, using physical contact via coaxial probes or resonant cavities. Of the non-contact methods employed, Orr et al., use microwave ground penetrating radar at 1.6 GHz to detect fluid presence within building sandstones and limestones, to provide calibration for moisture level tracking over long durations. Abdolrazzaghi et al. and Kazemi et al. use coupled complimentary split ring resonators to monitor variations in fluid composition and mixing with other fluids, such as water, methanol and ethanol, and for measuring glucose within blood samples, demonstrating the non-invasive value of microwave fluid detection, where the sample resides within a material that is not disturbed. Of the literature surveyed, two authors^64,65^ had applied their datasets to AI/ML processes for further processing and contrast identification, with insights gained on processing techniques.

**Table 1:** Comparison of literature using microwave sensing for fluid flow, fluid mixing, or geomaterial testing since 2018.

Ref-Year	Freq. (GHz)	Antenna type	Measurand	AI coupling?
Abdol-razzaghi et al.^64^	2.45	Coupled complimentary split ring resonators	Fluid type and mixing methanol, ethanol and water	Yes (DNN-CNN)
Kazemi et al.^65^	3.6	Split ring resonator and patch combination	Glucose within blood samples	Yes (LSTM)
Ferhat et al.^66^	0.8	VNA and contact probe	Hydration of cementitious materials	No
Shen et al.^67^	1–8	Wideband, non-resonant, coaxial leaky wave antenna	Water-cut monitoring	No
Alvarez et al.^68^	0.17–8.6	Multipoint coaxial re-entrant resonant cavity	Matrix property identification in liquid and powder geomaterial	No
Orr et al.^69^	1.6	Ground penetrating radar type (produced by Malå Geoscience)	Fluid detection within building sandstone and limestone	No
Blanche et al.^70,71^	24–25.5	Standard gain horn (characterized in Ref.^70^)	Fluid presence and extent if dry baseline is known An indication of mineralogy (CRIM and series/parallel models)	Yes (SVDD) for wind turbine blade health assessment^71^

The use of FMCW within the K-band for this work has been informed by the gains afforded by the higher frequency and improved resolution, in addition to the use of a highly directional standard gain horn antenna, in conjunction with the radar electronics, patent PCT/GB2017/053275, and with operating parameters shown in Table 4. This highly directional antenna was characterized in Blanche et al., where the effect of grain boundary geometries was also considered as a factor in return signal properties, with series and parallel models used to infer grain boundary angularity in addition to the complex refractive index method of geomaterial composition and the effect on permittivity^70^.

The justification for the use of K-band FMCW amounts to this method being non-contact, non-invasive and non-toxic at the power levels applied, in addition to being highly directional when coupled with a standard gain horn antenna. The microwave method is capable of both static and dynamic measurements to provide *right-time* data for further integration within cyberphysical systems and AI/ML processes, as exemplified in Refs.^64,65,71^.

Primarily a lab-based instrument and, though not necessarily more sensitive than other methods, such as NBT, XRT, contact tip and other free space microwave devices, FMCW provides a powerful, high resolution and sensitive alternative that does not require extensive preparation or large, costly equipment with limited access.

This paper presents the laboratory characterization of geomaterials with a static fluid content condition, where partial saturation with deionized water and kerosene are compared with air dry samples. Sandstone cores are subjected to dynamic load and induced failure events are monitored. Finally, a Frequency Microwave Continuous Wave (FMCW) sensor is used to detect water incursion of unsaturated porous sandstones. During FMCW monitoring, the sandstones used under these dynamic conditions have been loaded to failure prior to fluid injection. A control set of undeformed porous sandstone cores were also monitored to provide a comparison of porosity and permeability deviations due to loading and fracturing. Benchmarked against state-of-the-art geomaterial analysis methods, such as neutron beam and X-ray tomography, Scanning Electron Microscope (SEM) and Energy Dispersive X-ray (EDX) evaluation, we further demonstrate that FMCW offers a highly deployable, safe and economic alternative to the current state-of-the-art, with the potential to identify imminent sample failure in loaded sandstones. This will allow for greater control in loading a geomaterial sample up to, but not achieving, whole sample failure, allowing for greater control in the study of poroplasticity.

## Results

### Fluid and mineral presence in sandstones

To investigate the response of FMCW sensing to fluid and mineral presence, five porous geomaterials (Darney, Doddington, Lazonby, Locharbriggs and Red St. Bee’s sandstones^72–76^) were immersed in deionized water for one experiment and kerosene in a second experiment. Each immersion was for 19 h, resulting in a state of partial pore space fluid saturation as shown in Table 2^77^. Observations of the first interfacial FMCW return signal; the highest amplitude peak in the fast Fourier transforms, occurs at an intermediate frequency (IF) of 16 MHz, corresponding to a sample distance of 10 cm from the antenna, and with a decay tending towards the noise floor that is distinct for each partial saturation state. This section evaluates the variance in normalized reflection coefficient, $\Gamma$, due to partial fluid saturation tests.

**Table 2:** Pore-space occupancy values for partial saturation states for deionized water and kerosene after 19 h of immersion.

Sandstone sample	Water saturation (%)	Kerosene saturation (%)
Darney^72^	56.48	61.13
Doddington^73^	65.02	72.48
Lazonby^74^	54.14	61.28
Locharbriggs^75^	53.74	61.58
Red St. Bee’s^76^	60.46	65.22

**Figure 1:**
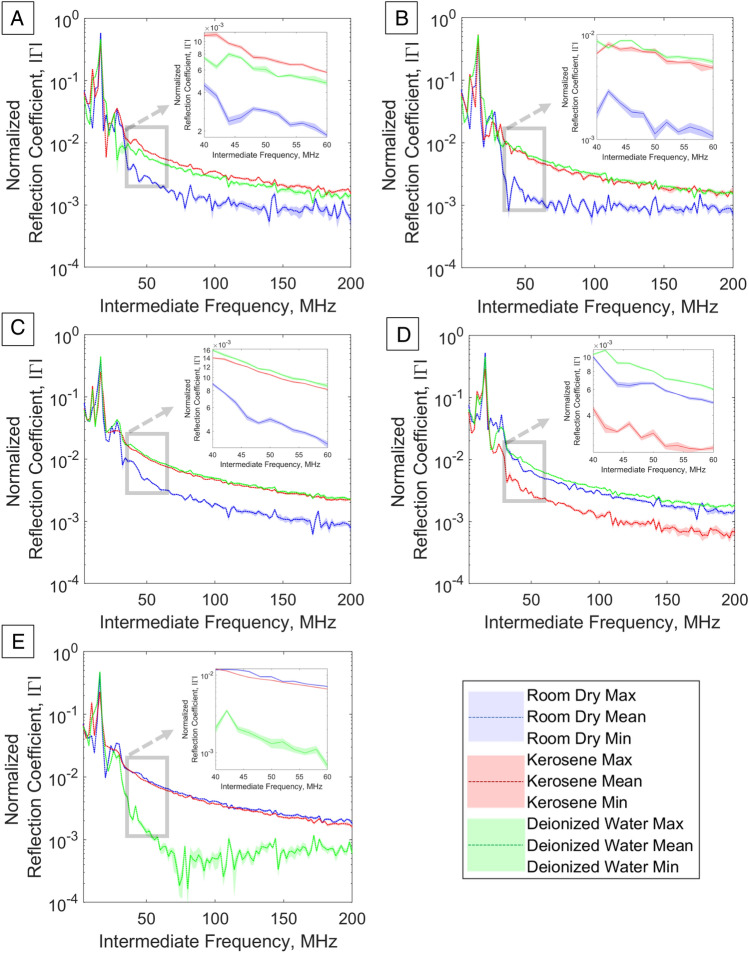
Fast Fourier transformed reflection coefficient waveforms in frequency domain for porous sandstones in a room dry (unwetted) state and with partial saturation with deionized water or kerosene. (**A**) Darney Sandstone, (**B**) Doddington Sandstone, (**C**) Lazonby Sandstone, (**D**) Locharbriggs Sandstone, (**E**) Red St. Bee’s Sandstone.

The plots for each sandstone and partial saturation state are shown in Fig. 1A–E, where the peaks in the asymptotic signal return are attributed to the many discrete interfaces found in the porous geomaterial. As this decay is representative of the bulk material, it may be utilized as a baseline for the unsaturated geomaterial and an indicator of the pore contents and internal properties of the geomaterial, when partially saturated.

### FMCW response to geomaterial deformation and failure under load

To investigate FMCW measurement sensitivity to loading of porous geomaterials, two sandstones were loaded via a computer controlled hydraulic press at a piston displacement rate of 187 mm hr^−1^, to the point of whole sample failure. The sandstones used were:

- Darney sandstone was selected for uniformity and homogeneity observed, as observed in rock characterization tests. This sample contains the least mineral and metallic inclusions^78,79^.
- Red St. Bee’s sandstone containing visible laminae of varying local hydraulic permeability, grain size, morphology and mineralogical composition^78,79^. This sample can be characterized as heterogeneous and anisotropic with regard to fluid permeability and clast properties.

**Figure 2:**
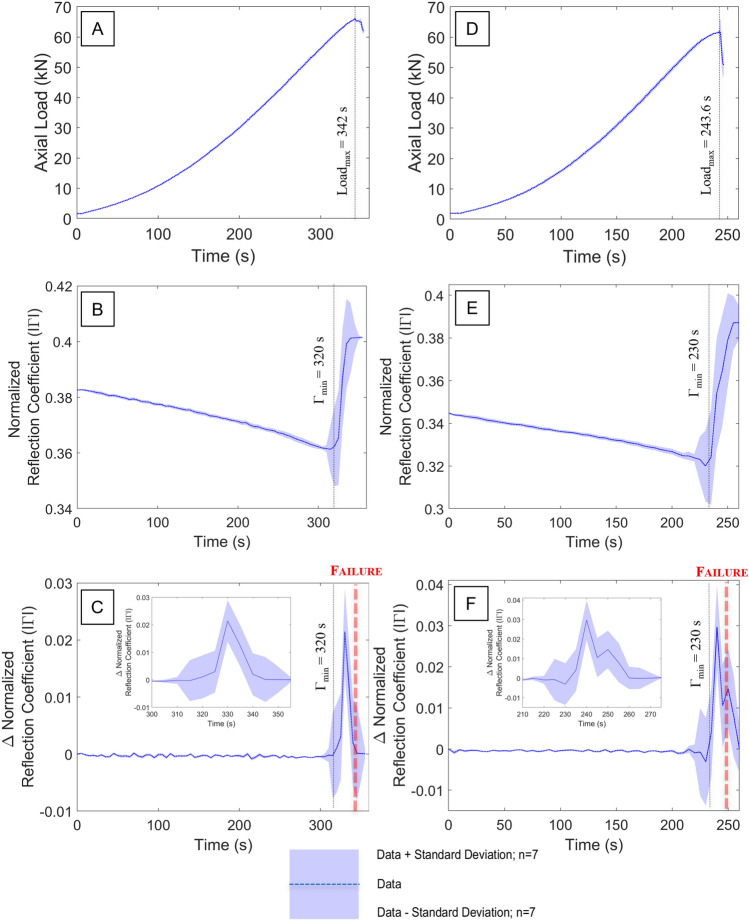
(**A**) Axial load vs. time for Darney 2B (**B**) reflection coefficient vs. time for Darney 2B (**C**) Rate of change in reflection coefficient vs. time for Darney 2B [inset focus on failure event] (**D**) Axial load vs. time for Darney 2 T (**E**) reflection coefficient vs. time for the Darney 2 T (**F**) Rate of change in reflection coefficient vs. time for Darney 2 T [inset focus on failure event].

The axial load vs. time plot for Darney sandstone sample 2B is shown in Fig. 2A, where the naming convention “2B” indicates that this sample was taken from the bottom of the second core cut from the Darney sandstone block acquired for this research. This protocol is to ensure that the samples are from adjacent areas within the cut block to minimise variation (facies changes, or subtle changes in manner of sediment deposition, for example). An axial force of 66.07 kN is defined as the peak load, ${{\text{Load}}}_{{\text{max}}}$, which occurs at 342.6 s. The minimum observed reflection coefficient ($\Gamma$), ${\Gamma }_{{\text{min}}}$, occurs at 315 s with a value of 0.361, a change of ~ 6% from the initial condition, ${\Gamma }_{0}$, of 0.383. Beyond this point, $\Gamma$ rapidly increases towards ${\Gamma }_{{\text{max}}}$ at 0.401 (Fig. 2B). The rate of change of $\Gamma$ is shown in Fig. 2C, where the sharp increase in the return signal can be observed as ${\Gamma }_{{\text{peak}}}$, which commences at 315 s and peaks at 330 s. A period of 12.6 s elapses between ${\Gamma }_{{\text{peak}}}$ and ${{\text{Load}}}_{{\text{max}}}$, indicating that the material failure as a response to load is the culmination of a series of events detectable by the FMCW sensor 15 s prior to reaching the ${\Gamma }_{{\text{peak}}}$ value, beyond which whole sample failure occurs.

The axial load vs. time plot for the Darney sandstone sample 2 T (top of second core) is shown in Fig. 2D, where sample failure was observed at a peak load of 61.82 kN, occurring at 242.7 s. ${\Gamma }_{{\text{min}}}$, occurs at 230 s (at 0.32) representing a change of ~ 7% from the initial condition, ${\Gamma }_{0}$, at 0.345 (Fig. 2E). Beyond this point, $\Gamma$ increases rapidly towards ${\Gamma }_{{\text{max}}}$, a value of 0.387 at 255 s. The rate of change in $\Gamma$ is shown in Fig. 2F, where the sharp increase in the return signal can be observed as ${\Gamma }_{{\text{peak}}}$, which commences at 230 s and peaks at 240 s. A period of 2.7 s elapses between ${\Gamma }_{{\text{peak}}}$ and ${{\text{Load}}}_{{\text{max}}}$. Compared to the Darney sample 2B, the lower ${\Gamma }_{0}$ value is interpreted to be due to the lower volume of high permittivity material within the field of view (FoV) of the FMCW sensor (Fig. 4B).

**Figure 3:**
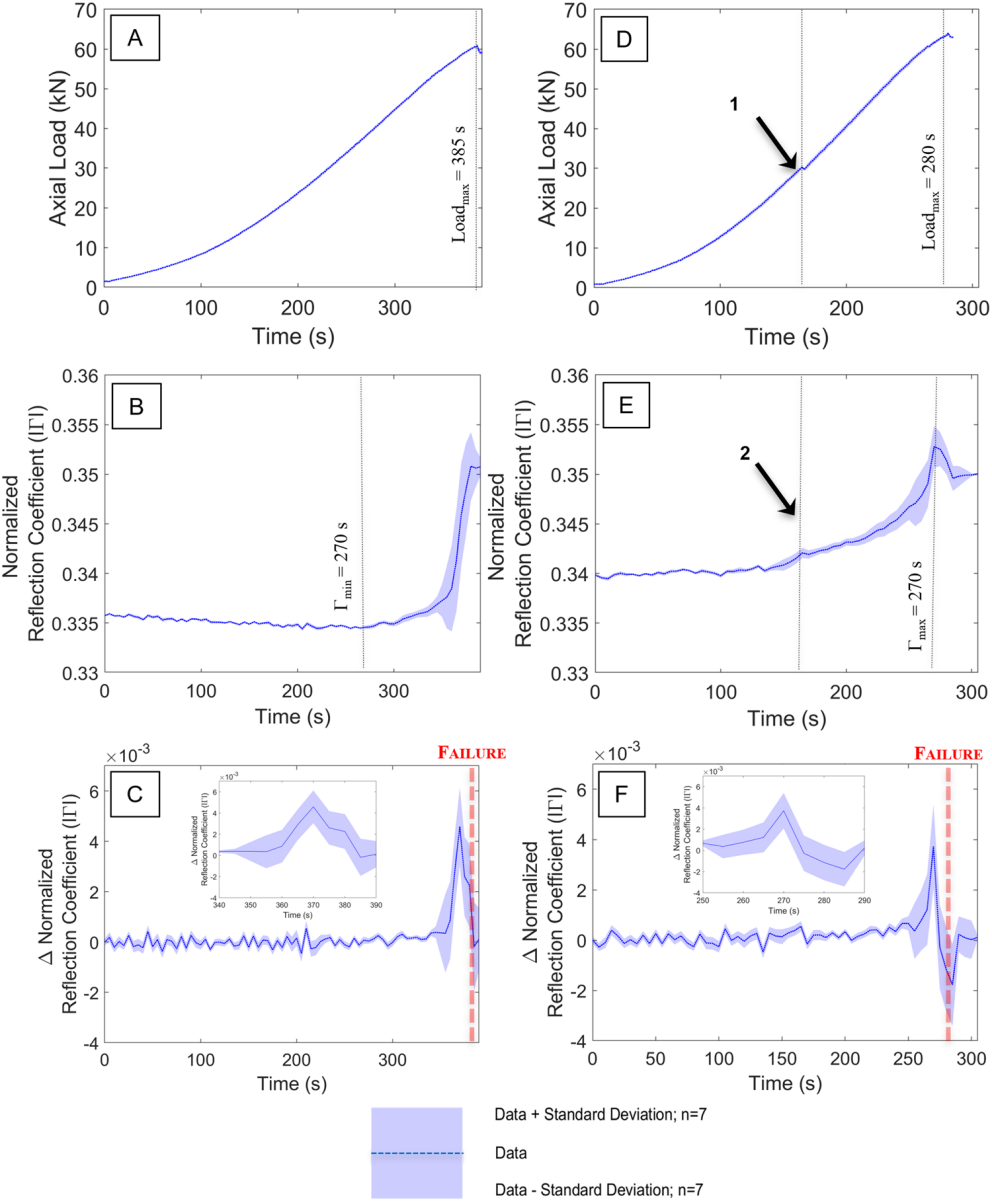
(**A**) Axial load vs. time for Red St. Bees 2B (**B**) reflection coefficient vs. time for Red St. Bees 2B (**C**) Rate of change in reflection coefficient vs. time for Red St. Bees 2B [inset focus on failure event] (**D**) Axial load vs. time for Red St. Bees 2 T [Point 1: region of load/slip associated with movement of laminae or fracture] (**E**) Reflection coefficient vs. time for Red St. Bees 2 T [Point 2: Change of slope due to FMCW detection of Point 1] (**F**) Rate of change in reflection coefficient vs. time for Red St. Bees 2 T [inset focus on failure event].

Figure 3A shows the axial load vs. time plot for the Red St. Bee’s sandstone sample 2B (bottom of second core). Sample failure is observed at 384.8 s, corresponding to a maximum load of 60.86 kN at. ${\Gamma }_{{\text{min}}}$ occurs at 205 s at a value of 0.334, a change of ~ 0.6% from the initial condition, ${\Gamma }_{0}$, of 0.336 Beyond this point, $\Gamma$ increases to a maximum of 0.351 at 380 s (Fig. 3B). The rate of change in $\Gamma$ is shown in Fig. 3C, where the sharp increase in the return signal can be observed as ${\Gamma }_{{\text{peak}}}$, which commences at 355 s and peaks at 370 s. A period of 14.8 s elapses between ${\Gamma }_{{\text{peak}}}$ and ${{\text{Load}}}_{{\text{max}}}$.

The axial load vs. time curve for the Red St. Bee’s sandstone sample 2 T (top of second core) is shown in Fig. 3D, where sample failure occurs at 280.4 s, corresponding to a peak load of 64.02 kN. ${\Gamma }_{{\text{min}}}$, occurs at ~ 10 s with a value of 0.34. Beyond this point, $\Gamma$ increases to a maximum of 0.353 at 270 s. A period of 260 s elapses between, between ${\Gamma }_{{\text{min}}}$ and ${\Gamma }_{{\text{max}}}$ (Fig. 3E). The rate of change in $\Gamma$ is shown in Fig. 3F, where the sharp increase in the return signal can be observed as ${\Gamma }_{{\text{peak}}}$, which commences at 255 s and peaks at 270 s. A period of 10.4 s elapses between ${\Gamma }_{{\text{peak}}}$ and ${{\text{Load}}}_{{\text{max}}}$.

A key differentiator for this sample is the gradient of the load rate, which starts with a positive slope and whereas all other tested samples maintain a negative slope during compression (Fig. 3F). This sample is also unique due to the event at 165.1 s (Point 1, in Fig. 3D), where sample resistance to the applied load is briefly interrupted. This suggests the coring process and/or the many laminae observed have already weakened the sample resulting in a partial failure or compaction/slip event at this point. The associated features observed within $\Gamma$ at the same timestamp (Point 2 in Fig. 3E) show a notable change in slope at ~ 165 s and a secondary peak value in the $\Gamma$ rate of change corresponding to partial sample failure (Point 3 in Fig. 3F).

### FMCW response to fluid flow and flood front detection

The sensitivity of neutron tomography allows excellent imagery of water movement through a porous geomaterial, where neutron interaction with the rock unit is minimal and enhances image contrast. The use of D_2_O (heavy water) and H_2_O as contrast agents also facilitates the tracking of water flow saturation processes, in addition to the delineation of water–air boundaries^12,69^. A major limitation in the neutron tomographic method is the requirement for extremely limited neutron source access and support facilities, resulting in limited experimental opportunities. Another limitation is the requirement for specialist sample preparation and the inability to deploy such large and expensive equipment for dynamic environment testing in the field. To investigate the sensitivity of the FMCW sensor to dynamic fluid ingress within a sealed, cylindrical sandstone core, we simultaneously exposed prepared samples of Darney and Red St. Bee’s sandstones to neutron tomography and FMCW radiation. This section presents the results of the combined neutron and FMCW data acquired at the Institut Laue-Langevin (ILL)^78^. Variations in the return signal amplitude, that are orientation dependent, are apparent during each sample rotation and are shown in Fig. 5, where FMCW return data was acquired during control measurements in the neutron beamline where each sample was rotated 180° during a period of 360 s. The parameters for the control measurements were the same as the fluid injection measurements except for the neutron tomography beamline. For the fluid injection phase of each experiment, an increase in $\Gamma$ is seen to correlate to the volume of fluid ingress within the samples and is observed in all samples.

**Figure 4:**
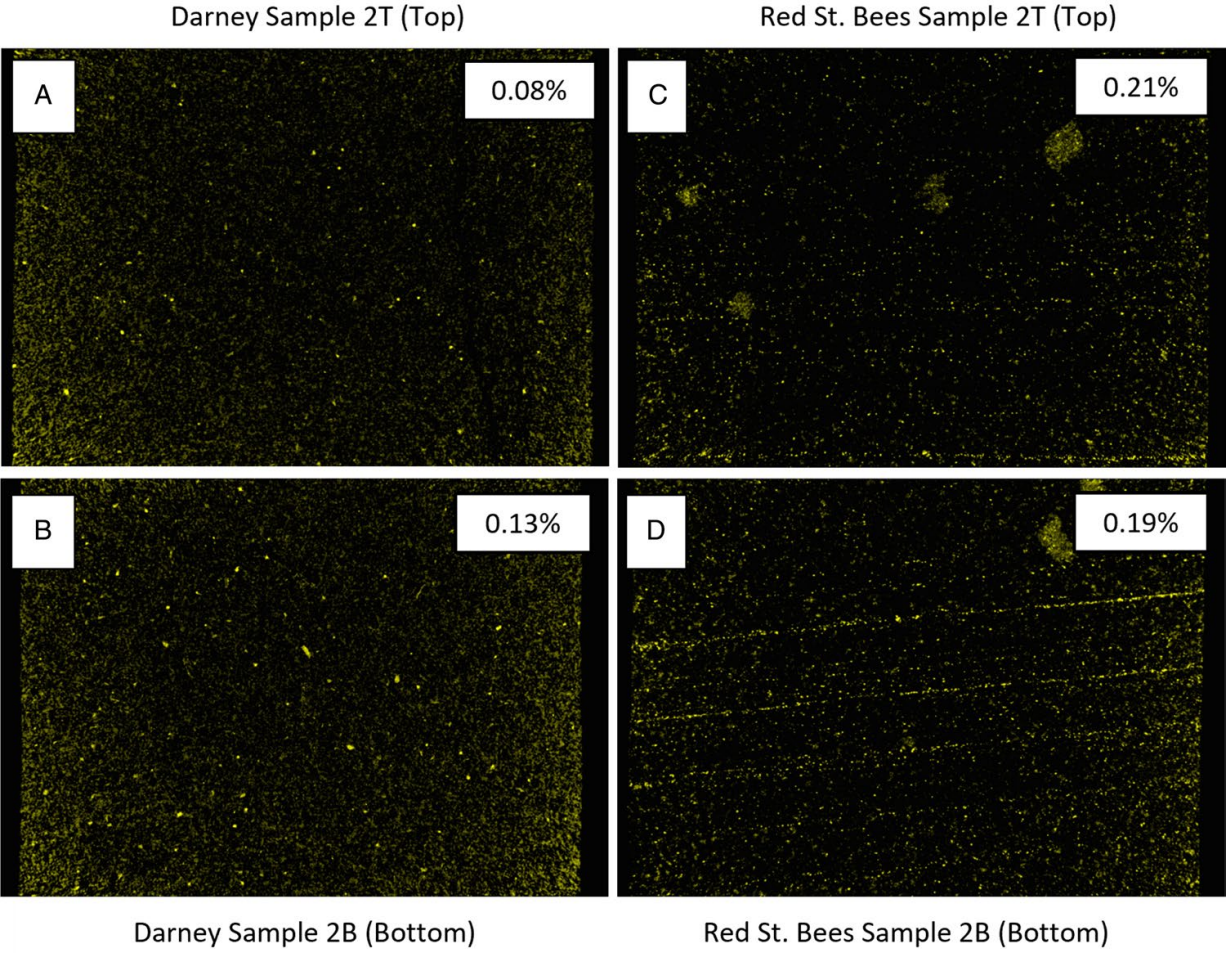
(**A**) Vertical X-ray reconstruction of high permittivity inclusions within the Darney 2 T sample with a volume content of 0.08% (**B**) Darney 2B sample with a volume content of 0.13% (**C**) Red St. Bees 2 T sample with a volume content of 0.21% (**D**) Red St. Bees 2B sample with a volume content of 0.19%. Each sample is 38 mm in diameter.

**Figure 5:**
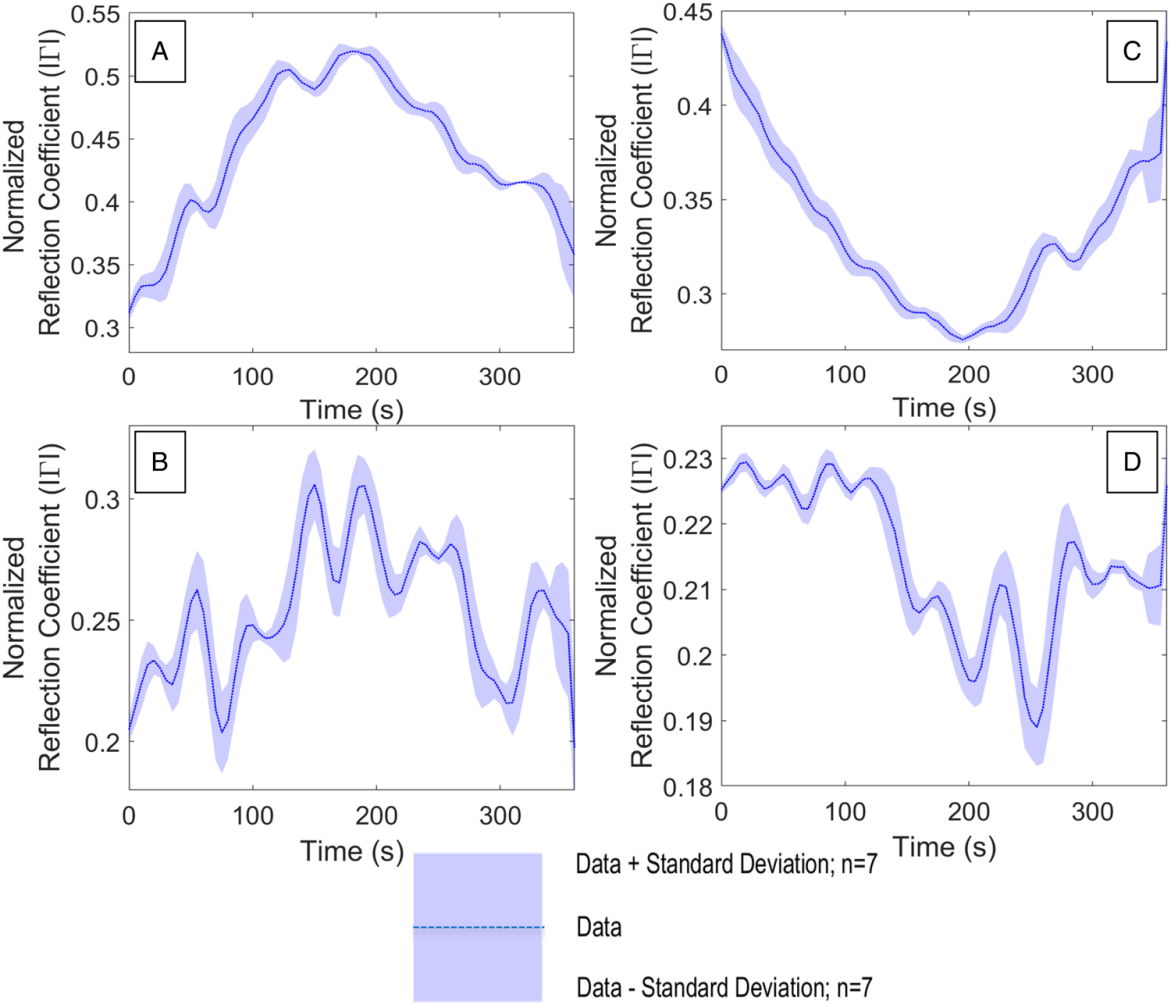
FMCW baseline measurements taken for one half rotation (180°) for each sandstone sample without X-ray, neutron beam or water injection active (**A**) Darney Sample 1BC (undeformed), (**B**) Darney Sample 2B (deformed), (**C**) Red St. Bee’s Sample 1TC (undeformed), (**D**) Red St. Bee’s Sample 2 T (deformed).

**Figure 6:**
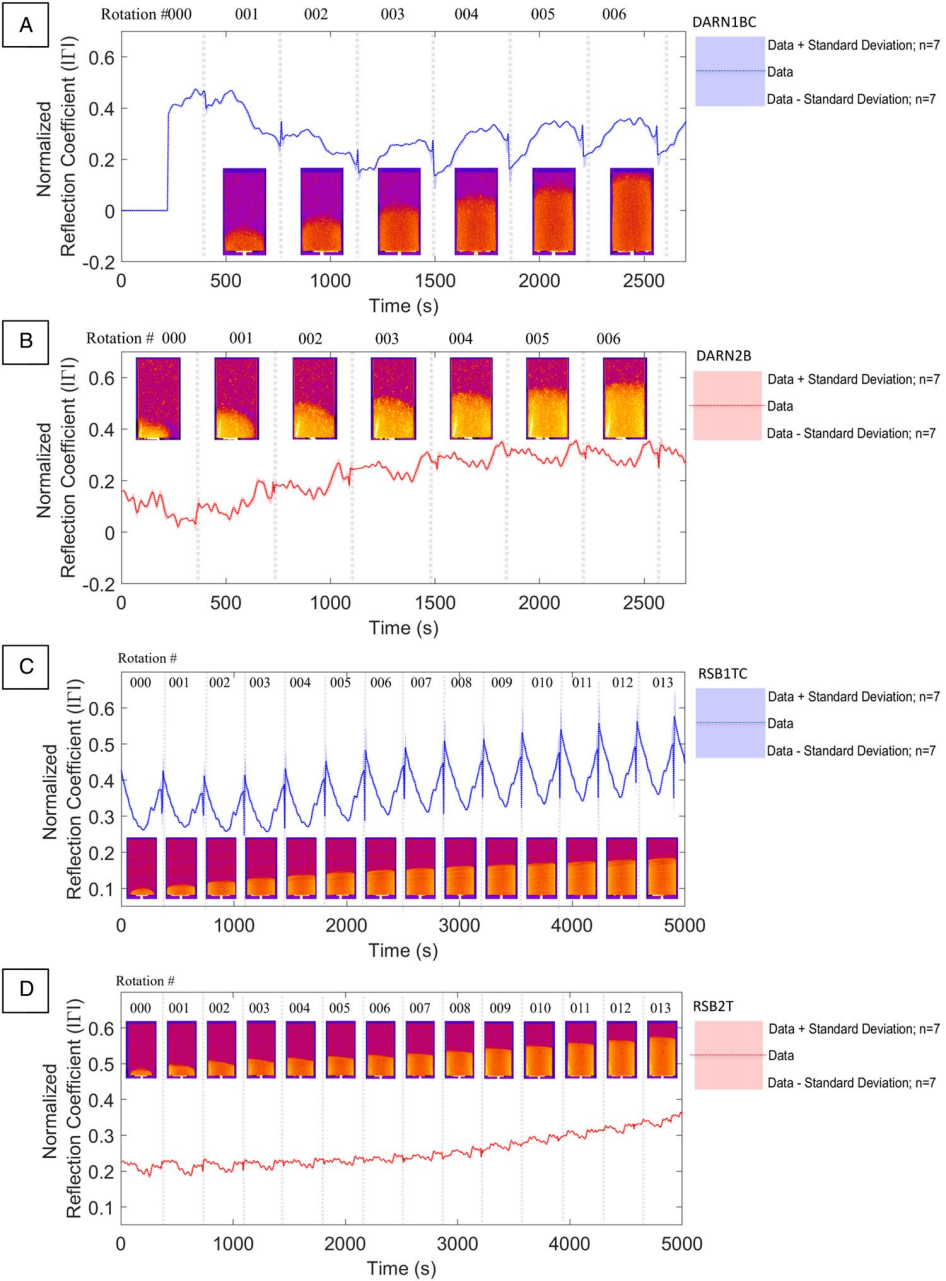
(**A**) (blue) Reflection coefficient vs. time for Darney 1BC (undeformed). (**B**) (red) Reflection coefficient vs. time for Darney 2B (deformed). (**C**) (blue) Reflection coefficient vs. time for Red St. Bee’s 1TC (undeformed). (**D**) (red) Reflection coefficient vs. time for Red St. Bee’s 2 T (deformed). Each half rotation is shown with neutron tomography acquisitions for the deionized water flood front progression (inset above and below data).

Figure 6 shows the correlations between the acquired neutron tomography images and where each 180° rotation would return to the start position before repeating. For the undeformed Darney 1BC sample, data acquisition spanned 8 consecutive half rotations in the beamline and totaled 45 min. It was found that FMCW data acquired for the first 225 s of rotation 000 was corrupt and not fully usable. Thus, the observed $\Gamma$ for rotation 000 initiated at ~ 225 s into the data acquisition process at a value of 0.377 and peaked at 0.475, prior to dropping to a value of 0.146 at the end of rotation 002. These high initial values are confirmed in the baseline FMCW test rotations, shown in Fig. 5, and are taken to be a characteristic of the unsaturated material property, rather than an error in measurement. Beyond rotation 002, a commensurate increase in $\Gamma$ may be observed with increasing deionized water volume uptake, evidenced in the neutron tomography imagery insets for each rotation in Fig. 6A, with the $\Gamma$ value observed to range between 0.23 and 0.378, dependent on orientation and corresponding to the maximum volume of partial water saturation in the sensor FoV. X-ray analysis of this sample allowed quantification of the highest relative permittivity constituent materials present, providing additional understanding of the distribution of metals and the mapping of magnesium, aluminum and iron-rich particles present. Adjusting the threshold of the imaging software^80^ used to generate the maps shown in Fig. 4, reveal that the percentage of high permittivity materials present within the FoV of the FMCW sensor is approximately 0.08% for the undeformed Darney sample (Fig. 4A).

The Darney 2B sample (Fig. 6B) data acquisition required 10 consecutive rotations, totaling 58 min in the neutron beamline. The datasets for this sample showed that, where the water was not within the FoV of the FMCW sensor, $\Gamma$ was between 0.012 and 0.155. This correlated with the baseline established in Fig. 5B and agrees with the high initial $\Gamma$ values observed for the Darney 1BC sample. As the water entered the FoV of the sensor, $\Gamma$ established a new minimum at 270 s with a $\Gamma$ value of 0.022 and steadily increased as water ingress continued to a maximum of 0.354, while continuing to vary with sample orientation.

Using the same analytical method employed for Darney sample 1TC, *ImageJ* software^80^ gives an approximate percentage of high permittivity materials within the FoV of the FMCW sensor of 0.13% (Fig. 4B).

The Red St. Bee’s 1TC sample (Fig. 6C) was exposed to 17 tomographies over 102 min of beam time. This sample shows the largest variance in $\Gamma$ due to orientation, with an initial range between 0.263 and 0.427 (Fig. 5C), a trend consistent with previous samples shows that the $\Gamma$ range decreases as the deionized water enters the sensor FoV. The $\Gamma$ range then consistently increases as the deionized water occupies more porosity within the FoV, peaking with a range of 0.377 and 0.596. This sample exhibits a lower hydraulic permeability than the Darney sandstone samples, evidenced by the longer fluid front transit time under the same injection rate conditions. However, the observed $\Gamma$ is consistent with the progression of the air–water front, as shown in the neutron tomography images. The use of *ImageJ* software^80^ with X-ray data determined that the percentage of high permittivity materials within the FoV of the FMCW sensor is approximately 0.21% (Fig. 4C).

The deformed Red St. Bee’s 1 T sample (Fig. 6D) was exposed to 14 tomographies, totaling 84 min in the beamline. The variation between the maximum and minimum return signal for this sample is the lowest of all the samples tested in this beamline experiment, with readings of $\Gamma$ (taken just as fluid uptake had commenced) ranging between 0.186 and 0.226. The continued influx of water from the sample base, resulted in a rise in observed $\Gamma$ to a peak range between 0.336 and 0.364. The percentage of high permittivity materials is approximately 0.19% for this sample (Fig. 4D).

## Discussion

### Static pore space occupancies in sandstones partially saturated with deionized water or kerosene

The observed trend is that the room dry samples return the lowest reflection amplitude, the partially saturated deionized water samples return the highest reflection amplitudes, with the partially saturated kerosene samples returning amplitudes that are close to, but less than, the deionized water samples.

Departures from this trend are:

- The Darney sandstone response when partially saturated with kerosene is higher in amplitude than the partially saturated deionized water sample. This may be due to differing partial saturation values for deionized water and kerosene, respectively.
- The Locharbriggs and Red St. Bee’s samples, when partially saturated with kerosene, return amplitudes are less than the room dry samples, this may be due to the high metallic mineral content increasing the bulk permittivity when unsaturated.
- The Red St. Bee’s partially saturated with deionized water has low $\Gamma$ and may be due to the relative abundance of high permittivity mineral inclusions increasing the bulk permittivity when unsaturated.

Table 3 provides data on EDX-acquired mineral compositions for each sample and shows that the Red St. Bee’s sandstone contains significantly more high permittivity metallic inclusions than the other sandstone samples.

**Table 3:** Mineral composition of sandstone samples acquired via EDX analysis.

%	Darney	Doddington	Lazonby	Locharbriggs	Red St. Bees
C	43.9	43.7	43.2	46.7	42.6
O	42.4	42.4	41.9	40.4	42.5
Si	12.9	13.4	13.8	11.6	12.2
Al	0.7	0.3	0.6	0.8	1.3
K	0.1	0.1	0.4	0.3	0.5
Fe	< 0.1	< 0.1	< 0.1	0.1	0.2
Mg	< 0.1	< 0.1	< 0.1	0.1	0.2
Ca	< 0.1	< 0.1	< 0.1	< 0.1	0.1
Na	< 0.1	< 0.1	< 0.1	< 0.1	0.2
Ti	< 0.1	< 0.1	< 0.1	< 0.1	~ 0.1

### Dynamic load on sandstone cores

The observed decreases in $\Gamma$ during axial loading are expected to be due to compaction within the loaded sample, where porosity reduction reduces the number of interfaces within the FoV of the sensor. The evolution of fractures due to compressive loading in porous geomaterials is well understood and highlights the role of microfracture formation, and consequent amalgamation into a macro-fracture, leading to sample failure^81,82^. By progressively loading the sample, open interfaces present within the sample reduce in width or close entirely. With the exception of the Red St. Bees sample 2 T, which is believed to have contained a macro-fracture prior to loading, this model explains the initial decrease in the $\Gamma$ observed as the axial load on each sample increased. The microfractures created as a result of increasing load shows a marked increase in planar interfaces within the sensor FoV resulting from granular failure and grain-grain cementation failure. As these samples were unconfined during loading, microfractures were expected align vertically throughout the sample, representing increased reflectance for microwave energy propagating at normal incidence to the microfractures, resulting in an observed increase in $\Gamma$.

The spatial resolution of the K-band, considered to be approximately half of the incident wavelength, is between 1.17 and 1.25 cm. Consequently, the response of the microwave FMCW sensor to a single hairline microfracture seems unlikely, and where the width of a microcrack is many orders of magnitude smaller in scale (nm to μm) than the incident wavelength^83^. However, the results presented within this research demonstrate that a consistent and detectable change in $\Gamma$ is observed as a function of applied load, with distinct signal contrasts observed during sample deformation up to 15 s in advance of irrecoverable yield, leading to whole sample failure.

### Dynamic fluid ingress into deformed and undeformed sandstone cores

**Figure 7:**
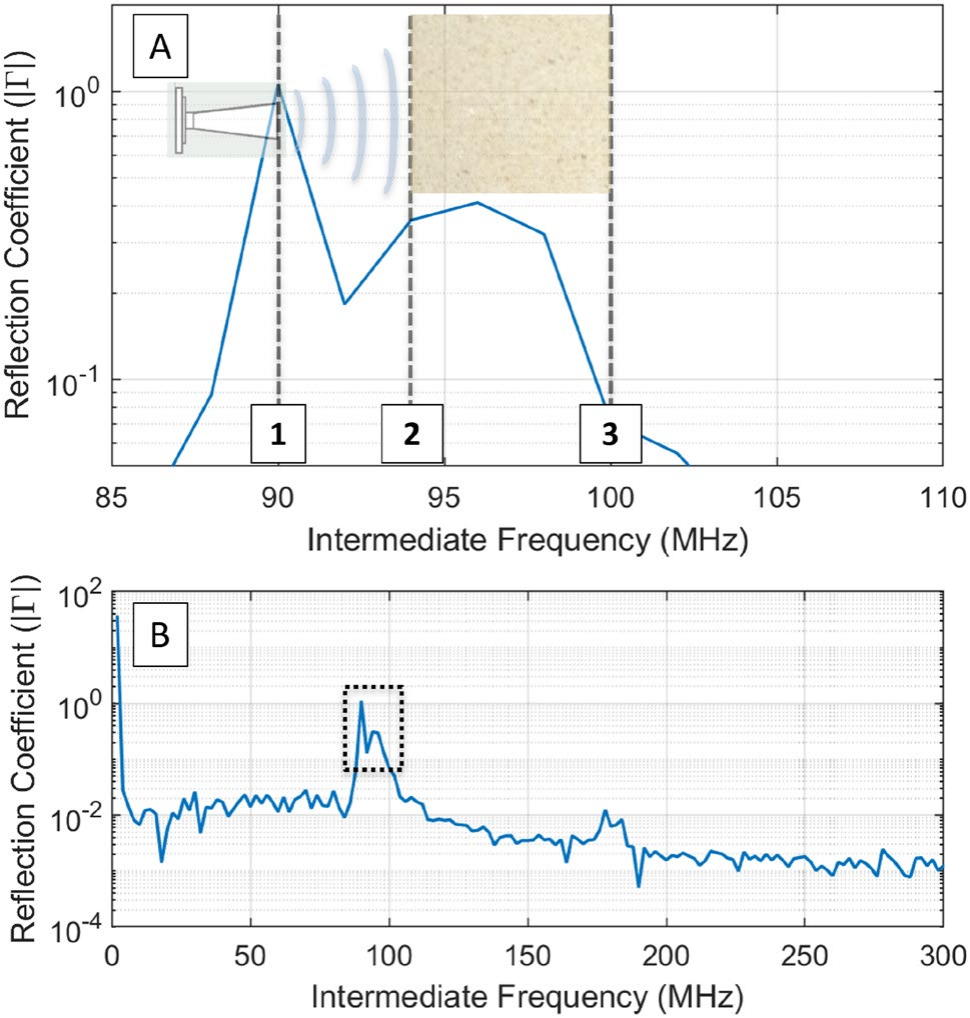
(**A**) Frequency domain transform of FMCW return data: point 1 is the antenna coupling, point 2 is the first sample interface (entry into sandstone cylinder) and point 3 is the second sample interface (exit from sandstone cylinder). (**B**) Shows the broader signal in the frequency domain, where the dashed line represents (**A**).

This research determines that FMCW is capable of observed ingress and progression of a deionized H_2_O fluid front during injection through the Darney and Red St. Bee’s sandstones. For all FMCW—neutron CT plots (Fig. 6), the reflections associated with the interior of the sandstone targets occur at an intermediate frequency of 96 MHz (see Fig. 7), with the return signal at this bandwidth corresponding to a region of rock volume extending some small distance within the sample (~ 1 cm). An evaluation of the Darney sandstone shows it to contain very few metallic inclusions (Table 3) and gives a signal return that may be indicative of a “clean” sandstone. The cyclic nature of the FMCW returns in all FMCW and neutron CT plots where the sample is rotating (Figs. 5, 6) are likely to be indicators of variations in:

- Clast orientation/bedding as a function of sample rotation, assuming FMCW is sensitive to crystal orientation.
- Position of high permittivity inclusions entering or leaving the bulk FoV within the sample during rotation.

For the Red St. Bee’s samples, the higher overall $\Gamma$ measurements for the undeformed sample versus the deformed sample may be due to an abundance of high permittivity inclusions present, which are not present in such abundance for the deformed sample, these are clearly visible in Fig. 4C as regions of high local metal content and are less prominent in Fig. 4D.

The results of this research demonstrate the capability of FMCW inspection for analysis of pore-space occupancies within partially saturated sandstones. Similarly, the FMCW modality may also be used for identification of geomaterial properties based on porosity variation and micro-/macro-crack presence, resulting from deformation and composition. The presence of deionized water ingress within the sandstone samples is clearly discernible.

## Conclusions

This application of non-destructive and non-invasive sensing shows that FMCW in the K-band can detect consistent signal contrasts to fluid presence, dynamic loading and fluid injection, where the advancing fluid position is represented via contrasts in the return signal properties within the sensor field of view. In demonstrating that FMCW in the K-band is sensitive to dynamic load up to sample failure, this new role for FMCW sensing will add to the set of characterization techniques currently used to observe fluid flow in geomaterials in laboratory conditions, with the added advantage that the FMCW technique is not restricted to specialist facilities. We demonstrate that FMCW sensing offers strong deployment potential and a reduction in experimental costs, while also demonstrating versatility in operation.

Improving our understanding of the subsurface will allow for optimized resource extraction or storage strategies, reducing expenditure and improving forecasting on loaded structures. While a trade-off exists in terms of resolution and penetration of the material when compared to higher powered and more complex, lab-based systems, we anticipate that FMCW represents a low cost and highly deployable sensing modality capable of identifying key features of interest in situ*,* and with the potential to act as a long-term residential sensor for early warning of major structural or petrophysical contrasts (loading, microcrack formation, oil–water contact migration, gas–water contact migration) in the field, when combined with cyberphysical systems, AI or ML. We demonstrate the potential for use as an improvement on geomechanical sensing for the study of poroplasticity in uniaxially loaded and deforming sandstones.

## Methods

### Static fluid and mineral presence in sandstones

This research utilized K-band FMCW module (patent PCT/GB2017/053275) in combination with Flann Microwave SGH antenna model #20240 and a K-Band rated (up to 26 GHz) 30 cm flexible waveguide (RG 402). Equipment parameters are given in Table 4.

**Table 4:** FMCW experimental parameters.

Parameter	Value (unit)
Band	K-Band (24–25.5 GHz)
Chirp duration	300 ms
Bandwidth	1500 MHz
Power consumption	40–900 mW 12 V DC supply
Range to target	10 cm
Acquisition frequency	0.2–1 Hz (tunable)

To prepare the room dry baselines, five differing sandstone types were pre-dried in an oven for 2 h at 60 °C and allowed to cool to room temperature at 20 °C. Samples were weighed to determine dry mass. The 10 × 10 × 1 cm samples were immersed in DI water or kerosene for 19 h and weighed to determine the partially saturated mass. The difference was calculated to give the mass of water contained^77^. Ten consecutive measurements were taken for statistical data at an antenna-to-sample distance of 10 cm. Reflection coefficient responses were plotted for the frequency domain via Hilbert Transform, following which amplitude extractions and Fourier transforms were applied. These data were normalized against metal analogue targets of similar geometry to the sample to provide a theoretical maximum value against which to plot $\Gamma$. In this section, a flat copper sheet was used at the same target separation from the antenna. A MATLAB code was written for continuous, sequential data acquisition (once per 5 s) and data processing focused on the key FFT IF numbers for interfaces and internal features. Observations of internal properties were inferred from contrasts in signal response beyond the established interfacial boundary observed at 10 cm from target.

For the calculation of saturation values:

1 $$m= \rho \times V \Rightarrow V= \frac{m}{\rho }$$

Using $\phi$ and $\rho$ from sandstone technical data sheets^72–76^, the volume including porosity can be calculated. Thus,

2 $$\left( {\frac{m}{\rho }{ }} \right) + \left( {\left( {\frac{m}{\rho }} \right){ } \times \emptyset_{{{\text{frac}}}} { }} \right) = V_{{{\text{solid}}}} { + }V_{\emptyset } = V_{{{\text{sample}}}}$$

If,

3 $$Partially \;saturated \;mass=Dry \;mass+Water \;mass$$

Then,

4 $$Water \;mass=Partially \;saturated \;mass-Dry \;mass$$

And,

5 $${V}_{water}= \frac{{m}_{water}}{{\rho }_{water}}$$

Therefore,

6 $$\% \;water\;saturation = \frac{{V_{water} }}{{V_{\emptyset } }} \times 100$$

### Geomechanical forces and deformation

Cores were taken from blocks of Darney and Red St. Bee’s sandstones to produce standard cylinders of 19 mm radius and 76 mm height. All sandstone samples, including control samples, were covered in a low-dielectric, heat-shrink polymer to prevent disintegration following fracture/failure.

The prepared samples were positioned in a computer-controlled hydraulic press. The FMCW antenna was positioned 10 cm from the sample, directed towards the sample mid-height, and the hydraulic press applied a load to maintain 187 mm h^−1^ of piston displacement. The start of the load sequence was synchronized with the start of radar acquisitions. The FMCW was set to the parameters given in Table 4 and data was taken every 5 s. Figure 7 exemplifies the frequency domain signal return following extraction from FFT, where the x-axis represents distance from the sensor. A 3 m co-axial waveguide allowed sufficient separation of the FMCW electronics modules from the mechanisms and support equipment near the hydraulic press, resulting in a sample interface peak at a higher IF value. Due to a right-angle connection bracket, the antenna is visible at 90 MHz (point 1). The first target interface is visible at IF = 94 MHz (point 2). 100 MHz (point 3) represents the exit interfaces of the sample. The region between 94 and 100 MHz is interpreted to represent the internal structure of the sandstone sample and where IF = 96 MHz represents an internal volume of the sandstone samples under load.

To calculate and plot $\Gamma$ values for IF 96 MHz, the IF output from the sensor was subjected to a Hilbert transformation to obtain the imaginary component of the complex signal. An amplitude extraction was then performed to arrive at the real component of the signal. An FFT of the dataset was performed to convert the time domain signal into the frequency domain. The same process was repeated for a metal analogue of the target to provide a theoretical maximum against which to plot $\Gamma$. In this section, the analogue was a cylinder of steel milled to the same dimensions as the samples. The $\Gamma$ values for IF 96 MHz were plotted as a function of time, where each consecutive data acquisition was at 5 s intervals. The difference between each consecutive data acquisition was calculated and output as the $\Delta\Gamma$ value for each geomaterial^84^.

### FMCW response to fluid flow and flood front detection

Previously deformed samples were taken to Institut Laue-Langevin (ILL) to test FMCW response to water injection while placed in the neutron beamline at experiment D50T site. The resultant neutron tomography was used to benchmark the FMCW response to the DI water injection. The longer co-axial waveguide (3 m) was used to allow sufficient separation of the FMCW electronics modules from the highly radioactive beamline. All samples, deformed and undeformed, were double jacketed (with high quality shrink wrap) to seal and prevent fluid bypass at sample edges. The FMCW antenna, and all other components exposed to the beamline, were covered in a graphite-rich absorbent material for protection. Observations of internal properties were inferred from contrasts in signal response beyond the established interfacial boundary observed at 10 cm from target (IF 96 MHz).

Eight sandstone standard cores (38 mm diameter × 76 mm height) were taken to ILL:

- Four Darney sandstone samples; two uniaxially deformed to failure and two undeformed control samples.
- Four Red St. Bee’s sandstone samples; two uniaxially deformed to failure and two undeformed control samples.

**Figure 8:**
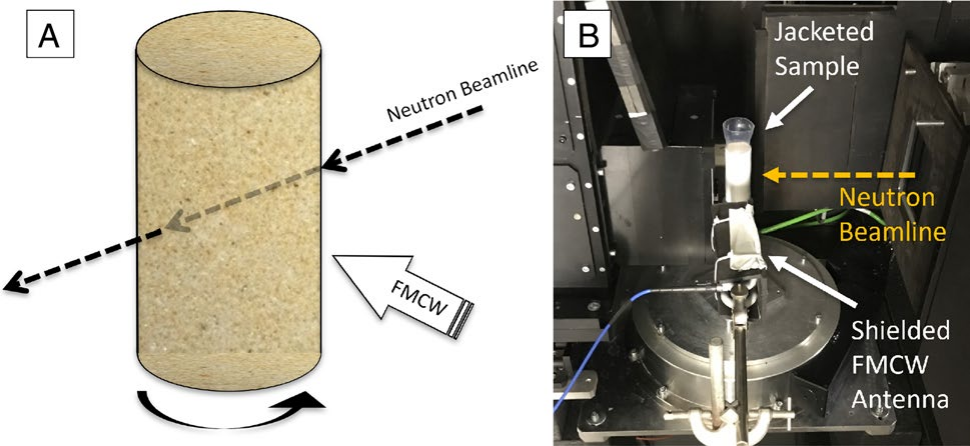
Schematic of the setup within the beamline. (**A**) Position of rotating sample with respect to the neutron beamline and FMCW incidence. (**B**) Sample in place within the beamline enclosure.

**Figure 9:**
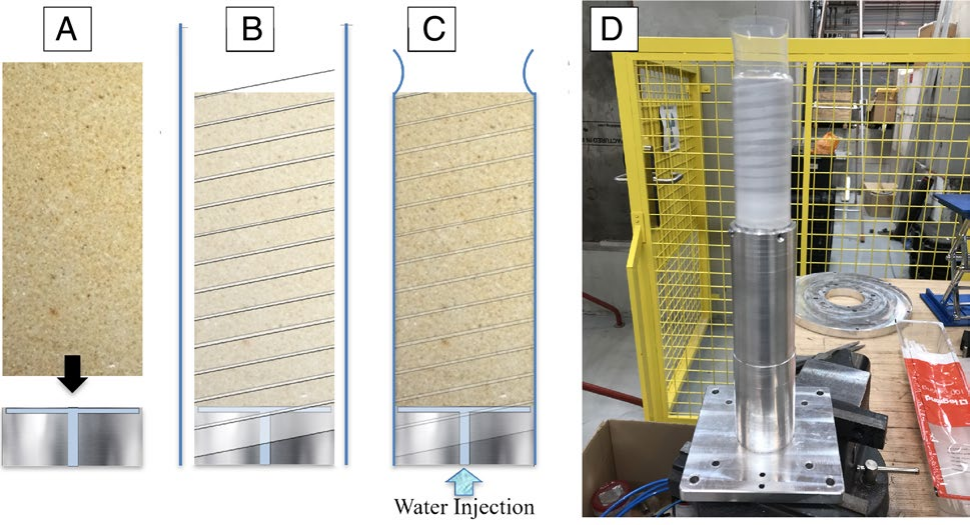
Technique used for mounting sandstone samples. (**A**) Sample is placed onto injection base of the same diameter. (**B**) Sample and base wrapped together in Teflon PTFE tape and a loose, heat-shrink jacket placed over assembly. (**C**) A heat gun was used to shrink the jacket, holding the assembly fast. The assembly is sealed, and water ingress is only possible via the injection port at bottom of sample. (**D**) The mounted sample prior to fluid injection and imaging^78^.

The experimental setup required synchronous recording of neutron and FMCW data, with orthogonal incidences for each detector (Fig. 8A,B). The base of each jacketed sandstone sample was injected at a controlled rate of 0.4 mm h^−1^ with deionized water and excess water collected in a reservoir above the sample. The methods employed for these tests are fully described in Lewis, Couples^85^, where the cup assembly included an aperture at the base containing a small reservoir, allowing the regulation of fluid flow from the base of the sample upwards (Fig. 9A). The sandstone core was positioned onto the cup and reservoir assembly and both the sample and cup were wrapped in PTFE/Teflon tape (Fig. 9B) and jacketed in a heat-shrink polymer to ensure sample was both sealed and affixed to the mounting (Fig. 9C,D).

The combination of shrink-wrap and PTFE tape was found to be effective to prevent fluid loss and ensured the cylinder sides were no-flow boundaries. The shrink-wrap also provided added stability when wrapped around the metal plinth supporting the sample, as correction of any unwanted movement in the radioactive beamline would be impossible.

The jacketed assembly was then placed in the beamline chamber, centered on the rotation stage and fixed in place.

All experiments were conducted at ambient temperature (~ 20 °C) with the FMCW antenna placed out of line of the neutron beam, 10 cm from the sample surface and in a position shielded from the neutron beamline. The FMCW waveguide used for these experiments was a 3-m Pasternack flexible coaxial cable to allow significant distancing of the FMCW electronic modules from the radioactive area. This adaptation from the usual experimental setup, using a 30 cm coaxial waveguide, results in a longer two-way pathlength for the signal to the target. For this reason, all experimental data used for this test was taken from IF 96 MHz; identified as the first interfacial reflection of the sandstone targets in the FMCW FoV. All tests used deionized water, with a controlled injection rate of 0.5 ml/min, resulting in a sample base water pressure of approximately 10 kPa. Each test took approximately 6 h to prepare and perform, with several hours required to allow for radioactive decay to return to a safe level.

All tests in the neutron beamline followed the sequence below:

- X-ray radiography data was acquired with no introduced water. This sequence provided a representation of the distribution of material within the sample, highlighting any major features like fractures and large-scale, high permittivity inclusions (e.g., metallic minerals).
- One half rotation of neutron radiography with FMCW running to determine the radar sensitivity to orientation and to set a dynamic baseline for each sample. These measurements provide insight on the expected radar return signal range as a function of rotation while the neutron beamline is active.
- Introduction of water at sample base, acquiring neutron radiography with simultaneous FMCW for consecutive half rotations. All tests with injected with H_2_O. These tests allow for the tracking of fluid though the sample using the full field method and to test the FMCW sensor for flood front sensitivity.

The ILL tech team took continuous neutron scattering data via scintillation detectors, which were compiled via computerized tomography to generate 3D models of the water distribution during injection within the sample. X-ray data was also acquired for high permittivity material distribution/mapping within the samples^86^. FMCW response was measured during fluid injection process to determine the progress of the flood front through the sample for comparison with the neutron CT data. The parameters of data acquisition used were the same as for the dynamic load measurements, with a 5 s chirp interval. The method used to interpret dynamic internal fluid flood front passage within the FoV of the sensor was also the same as the previous section, with $\Gamma$ peak corresponding to IF 96 MHz.

### Measurement consistency

Understanding signal variation as a function of operation and experimental runtime is crucial to data fidelity, especially during prolonged use of the FMCW hardware. An investigation was carried out to test the thermal output of the hardware, to ensure reliability and confidence in returned data as a function of experimental duration. The following test had a duration of 5500 s of continuous data acquisition, with a 300-ms chirp emitted once every 5 s. Key parameters are as follows:

- Flann antenna (model: #21240-20/serial: #219405)
- Pasternack 3 m K-band rated waveguide
- Antenna distance to target 100 mm
- Target Steel reference cylinder (38 mm diameter and 130 mm height)

**Figure 10:**
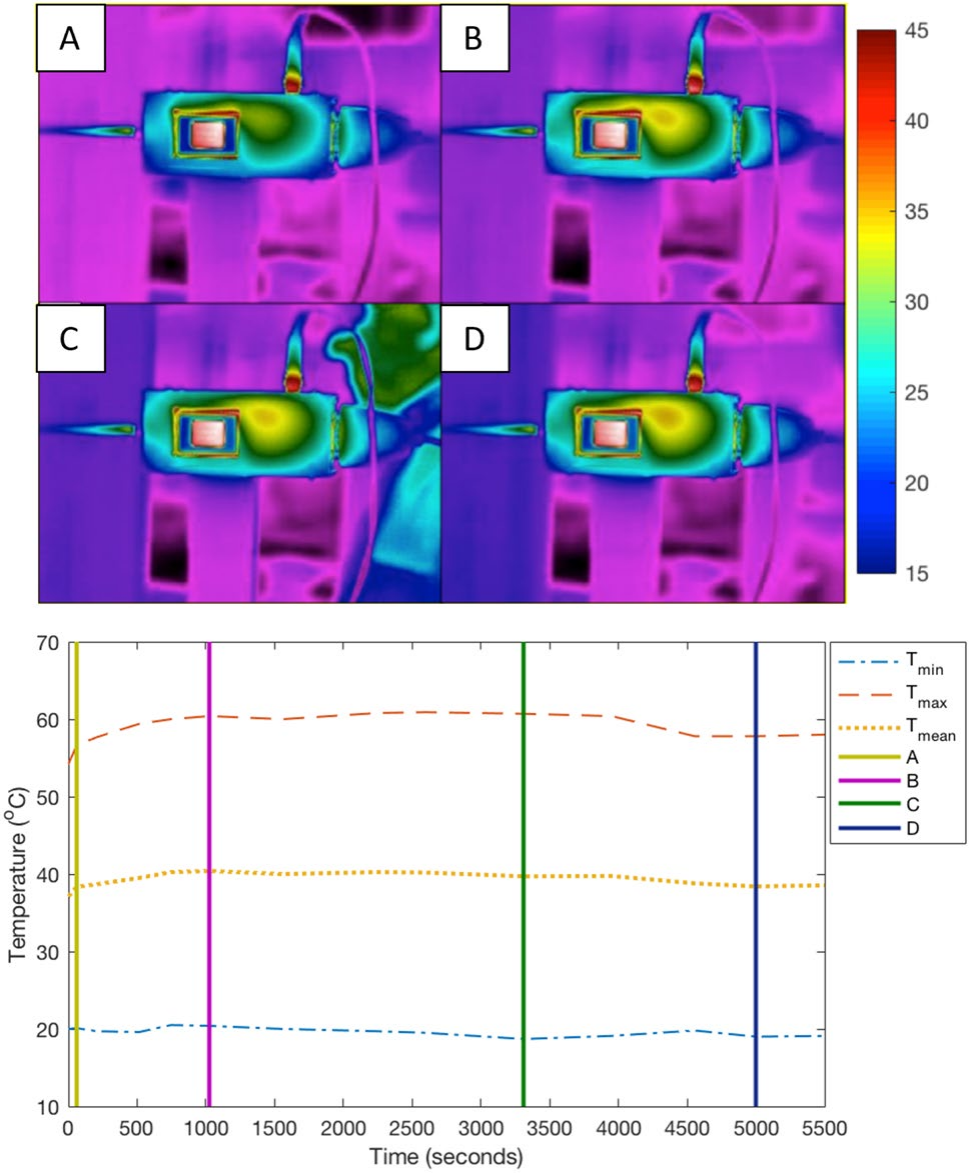
K-Band FMCW module and control board temperature during 5500 s of continuous operation. (**A**) 10 s, (**B**) 1000 s, (**C**) 3300 s, (**D**) 5000 s. Scale bar in ^o^C.

Throughout the duration of this test, the electronics module was photographed using a FLIR A300 Series (Serial #429100418), with the temperature variation of the electronic modules recorded. Figure 10 (top) shows the K-Band FMCW module and control board temperature during the 5500 s of continuous operation. A: 10 s, B: 1000 s, C: 3300 s, D: 5000 s. Scale bar in °C. Figure 10 (bottom) displays the thermal variation of the sensor and surroundings as a plot with clear consistency in temperature while operating with the experimental parameters used for this research.

### Statistics

The loading to failure of each sample resulted in datasets unique to each loaded core and the experimental process required iterations that were non-repeatable for a particular sandstone core. The provision of error bars for the resultant plots were derived from the calculation of a moving standard deviation for each dataset with a sample size (n) of 7. This moving standard deviation was added to, or subtracted from, the original dataset to provide an upper and lower error range. This method was applied to all datasets where the FMCW measurement could not be repeated.

### Supplementary Information

Supplementary Information 1.

Supplementary Information 2.

Supplementary Information 3.

Supplementary Information 4.

Supplementary Information 5.

Supplementary Information 6.

Supplementary Information 7.

Supplementary Information 8.

Supplementary Information 9.

Supplementary Information 10.

Supplementary Information 11.

Supplementary Information 12.

Supplementary Information 13.

## Data Availability

All data generated or analyzed during this study are included in this published article (and its Supplementary Information files).
